# Estimation of Target Hazard Quotients and Potential Health Risks for Toxic Metals and Other Trace Elements by Consumption of Female Fish Gonads and Testicles

**DOI:** 10.3390/ijerph19052762

**Published:** 2022-02-27

**Authors:** Kamila Pokorska-Niewiada, Agata Witczak, Mikołaj Protasowicki, Jacek Cybulski

**Affiliations:** Department of Toxicology, Dairy Technology, and Food Storage, Faculty of Food Sciences and Fisheries, West Pomeranian University of Technology in Szczecin, Papieża Pawła VI Street 3, 71-459 Szczecin, Poland; agata.witczak@zut.edu.pl (A.W.); mikolaj.protasowicki@zut.edu.pl (M.P.); jacek.cybulski@zut.edu.pl (J.C.)

**Keywords:** toxic metals, trace elements, freshwater fish, health risk assessment, fish consumption

## Abstract

The aim of this study was to assess the risk to consumers associated with the intake of toxic metals and other trace elements in diets that include the female gonads, testicles, and muscles of four popular freshwater fish species in Poland—common bream (*Abramis brama* L.), European perch (*Perca fluviatilis* L.), common roach (*Rutilus rutilus* L.), and northern pike (*Esox Lucius* L.). The following methods were used to determine the elements: GF-AAS (Pb, Cd); CV-AAS (Hg); ICP-AES (Zn, Fe, Mn, Cu, Ni, Li, Cr, Al). The concentration of toxic elements (Hg, Cd, Pb) in the female gonads and testicles ranged from 0.004 ± 0.006 mg/kg (Cd) to 0.028 ± 0.018 mg/kg (Pb). Of the other elements, the lowest content was noted for Cr (0.122 ± 0.182 mg/kg) and the highest for Al (46.98 ± 31.89 mg/kg). The study confirmed that female gonads and testicles are a valuable source of essential trace elements (Zn, Fe). Considering the content of toxic elements, the raw material of female gonads and testicles posed no health risks (THQ < 1).

## 1. Introduction

Fishes are a valuable human food thanks to the high contents of omega 3 polyunsaturated acids, fat-soluble vitamins, and essential minerals [1]; however, fishes readily take up contaminants, including trace elements, especially toxic metals (cadmium, lead, and mercury), from the water and food and accumulate them in their tissues [2,3].

Until recently, fish gonads were considered to be fish waste products, but currently, they are increasingly being used as food. Caviar, or sturgeon eggs, is known worldwide. It is considered to be a potential therapeutic product [4], and it is thought to have an advantageous influence on cardiovascular diseases, colon cancer, chronic inflammation, cognitive disorders, and immunomodulation. Testicles are used mainly in Asian cuisines and are considered to be an aphrodisiac. Most of these health claims require confirmation through clinical trials [5]. The eggs of fishes other than sturgeons, in addition to serving as caviar substitutes, are consumed dried or fermented, for example, in Russia, Japan, and Scandinavia [6]. It is assumed that thanks to the physiological function they serve, female fish gonads and testicles can accumulate valuable microelements that can be utilized in the production of functional foods. There are, however, few data on the proximate composition of fish gonad products, especially on the content of both essential and toxic trace elements. The proximate composition of female fish gonads and testicles depends on species and environmental factors and also on the stage of gametogenesis [7].

To determine the threat to human health stemming from the intake of carcinogenic and non-carcinogenic elements with portions of fish, the U.S. Environmental Protection Agency (USEPA) introduced the target hazard quotient (THQ) and hazard index (HI). These coefficients are used to assess potentially non-carcinogenic threats to health [8]. USEPA (2000) also recommends calculating the maximum allowable fish consumption rates (e.g., EDI—estimated daily intake and PTWI—provisional tolerable weekly intake) to minimize carcinogenic and non-carcinogenic effects on health [9,10].

The aim of the study was to determine the possibility of using products from freshwater fish gonads as sources of essential micronutrients in the human diet and to determine the potential risk associated with the consumption of toxic metals accumulated in them. The contents of the elements found in the female fish gonads and testicles are presented in comparison with the contents of elements in muscle tissues.

## 2. Materials and Methods

### 2.1. Sample Collection and Preparation

**Figure 1:**
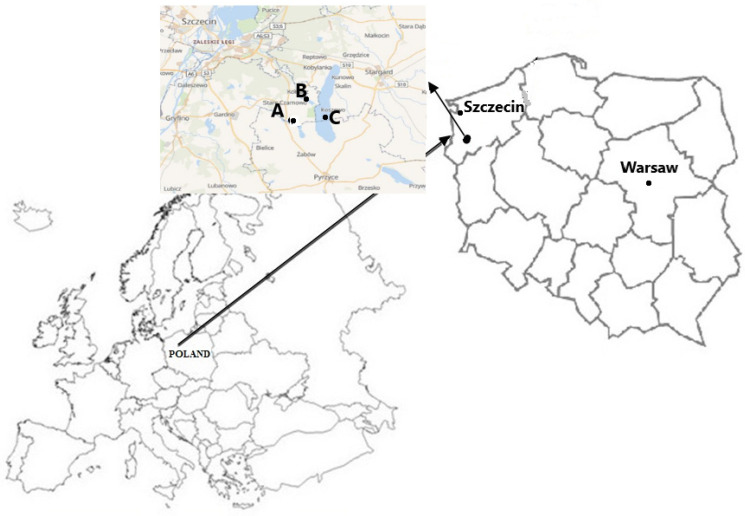
Location of the study area. Notes: A—Płoń Lake; B—Żelewko Lake; C—Miedwie Lake.

The study materials were collected from three lakes in the West Pomeranian Voivodeship (Poland) (Figure 1). The fishes collected for the analysis came from lakes Miedwie, Płoń, and Żelewko. It is an area of habitat protection, the main purpose of which is to protect habitats of various species of fauna and flora, which are protected or significant for the ecosystem of the region [11].

The catchment area of the Miedwie, Płoń, and Żelewko lakes consists mainly of agricultural areas where nitrogen fertilizers are applied. The quality of lake sediments is systematically controlled taking into account geochemical [12] and ecotoxicological criteria [13]. The values of all analyzed elements met the geochemical criteria for class I sediments (uncontaminated sediments) and were (mg/kg): Ag < 1.0, As < 10, Cd < 1, Cr < 50, Cu < 40, Hg < 0.2, Pb < 30, Ni < 16, Zn < 200 [12]. The investigated area met the ecotoxicological criteria also at level I, i.e., not exceeding the following values [mg/kg]: Ag < 1.6, As ≤ 9.8, Cd ≤ 0.99, Cr ≤ 43.0, Cu ≤ 32.0, Hg ≤ 0.18, Pb ≤ 36.0, Ni ≤ 23.0, Zn ≤ 120.0 [13,14]. Samples were collected from March 2018 to February 2020. Samples for the study included four fish species; two were predatory—European perch (perch, *Perca fluviatilis* L.) and northern pike (pike, *Esox lucius* L.)—and two were non-predatory—common roach (roach, *Rutilus rutilus* L.) and common bream (bream, *Abramis brama* L.). These species differed in their diet. Bream: -larvae and fry—plant and animal plankton; -juveniles, initially with plankton and then with benthic food (insect larvae, oligochaetes, mollusks, and plant debris). Roach: -fry and juveniles—algae, planktonic crustaceans, insect larvae, detritus; -older—insect larvae, vascular plants, mollusks, to a lesser extent plankton crustaceans. Perch: -juveniles—plankton and insect larvae; -from 7 cm in length, they begin to lead a predatory lifestyle; adults—predators. Pike: feeds mainly on fish, exceptionally large individuals hunt other vertebrates (frogs, birds, and mammals) [15]. The condition of the fish was determined on the basis of Fulton’s condition factor (K), which was calculated as follows: K = (100 × Fish weight (g)) × Fish length (cm)^−3^. A value above 1.20 indicates very good fish condition, within the range 1.00–1.20 fish good condition, and values below 1.00—poor condition of fish (in extreme cases—emaciated or sick fish) [16]. Table 1 presents the biometric data of the fish examined.

Immediately after being caught, the fish were transported to the laboratory on ice in containers. Fish total length and weight and gonad size were measured (Table 1). Samples of female fish gonads, testicles, and muscles (skinned) with the dorsal part, were excised with a stainless steel knife, packed in labeled, resealable polyethylene bags, and stored at a temperature of −30 °C until analyses. When purchased from fisheries enterprises, the fish used in the study were dead. In accordance with European and Polish legislation, studies of tissues and organs from fish caught for commercial sale do not require obtaining permission from the Local Ethics Commission.

### 2.2. Analysis of Female Fish Gonads, Testicles, and Muscles

Female gonads, testicles, and muscle samples of 1 g ± 0.001 g were collected for the analysis of trace elements. The samples were digested with an MDS-2000 microwave mineralization system (CEM Corp., Matthews, NC, USA) with 3 mL of concentrated HNO_3_ (Suprapur, Merck KGaA, Darmstadt, Germany). After cooling, the samples were filtered into polyethylene bottles and diluted with deionized water to a volume of 25 mL (0.05 μS/cm Barnstead™ GenPure™ Pro, Thermo Scientific, Hennigsdorf, Germany). The microelements were determined with inductively coupled plasma-atomic emission spectrometry in a Jobin Yvon JY-24 apparatus equipped with a Meinhard TR 50-C1 (ICP-AES) nebulizer. Cd and Pb contents were determined with the flameless atomic absorption spectrometry with electrothermal atomization in a graphite cuvette with Zeeman background correction (GF-AAS) in a Perkin Elmer ZL 4110. Mercury content was determined with cold vapor atomic absorption spectrometry (CV-AAS) in a Bacharach Coleman MAS-50 mercury analyzer.

### 2.3. Analysis Quality Assessment

Analytical method quality was verified based on limits of detection (LOD) and quantification (LOQ), recovery, and precision. LOD and LOQ were determined with standard deviations of blank samples multiplied by three and six, respectively. The limits of LOD and LOQ (μg/L) were as follows: Zn—1.6, 7.4; Ni—0.01, 0.04; Fe—4.2, 8.6; Mn—1.3, 4.1; Cr—0.06, 0.2; Cu—5.0, 10.1; Pb—1.02, 3.10; Cd—0.091, 0.215; Hg—0.1, 0.29. The quality of determinations was verified every 12 samples based on calibration coefficient values. The assumed calibration coefficient value limit was ≥0.995. The precision of the analytical procedure applied was verified with MODAS-3 certified reference material (MODAS Consortium, Institute of Nuclear Chemistry and Technology, Warsaw, Poland). The recovery values and coefficients of variation (CV) for the elements analyzed were as follows: Zn—93.1%, 5.8%; Ni—97.5%, 3.3%; Fe—94.9%, 3.9%; Mn—97.1%, 3.1%; Cr—94.1%, 4.5%; Cu—98.5%, 3.4%; Pb—97.5%, 3.9%; Cd—97.0%, 3.5%; Hg—95.4%, 2.8%.

### 2.4. Calculation of Micronutrient Uptake

The weekly supply of microelements from gonads was calculated assuming an adult weighing 70 kg consumes 34.8 g/day of the above products [17]. Unfortunately, in Poland, there is no information on the consumption of fish gonads, as it is a product that is not widely used, so the average consumption of fish muscles and fish products was referred to. Based on the average values in female and male gonads, the percentage coverage of the daily requirement for micronutrients for an adult was determined. The recommended dietary allowances were as follows: zinc and iron—8 mg/day for women and 11 mg/day for men; copper—0.9 mg/day; lithium—1 mg/day; manganese (AI)—1.8 mg/day for women and 2.3 mg/day) [18].

### 2.5. Risk Assessment to Human Health

The risk assessment to humans was based on the following parameters:

1. Estimated Daily Intake (EDI) (Equation (1)) [8,19].
  (1) $$\mathrm{EDI}=\frac{\mathrm{MS}\cdot C}{\mathrm{BW}}[\mu g/\mathrm{kg}\;\mathrm{bw}/\mathrm{day}]$$
  where: MS—the daily food ingestion rate in grams per day 34.8 g/day [17]; C—fresh weight concentration of trace elements in fish muscles, female gonads, and testicles (mg/kg); BW—reference body weight of 70 kg.
2. Target Hazard Quotient (THQ) (Equation (2)) [9,20].
  (2) $$\mathrm{THQ}=\frac{\mathrm{EF}\times \mathrm{ED}\times \mathrm{MS}\times C}{\mathrm{RfD}\times \mathrm{BW}\times \mathrm{AT}}\times 10^{-3}$$
  where: EF—exposure frequency to trace elements (365 days/year); ED—exposure duration (70 years); MS—food ingestion rate, 34.8 g/day [17]; C—concentration of trace element in fish muscles and female gonads and testicles (mg/kg); RfD—oral reference dose of trace element (mg/kg BW/day) (Zn = 0.3; Ni = 0.02; Fe = 0.7; Mn = 0.14; Cr = 0.003; Cu = 0.04; Li = 0.02; Pb = 0.0035; Cd = 0.001; Hg = 0.0001) [3,9]; BW—reference body weight of 70 kg; AT—averaged exposure time e to non-carcinogenic trace elements (365 days × 70 years).
3. Total Target Hazard Quotient (TTHQ) (Equation (3))—total THQ of all elements analyzed [21,22].
  (3) TTHQ=THQ(Zn)+THQ(Ni)+⋯THQ(Hg)
4. Provisional Tolerable Weekly Intake (PTWI) (Equation (4))

Values were multiplied by the average adult body weight (BW—70 kg). Then, the percent PTWI was calculated [23,24].

(4) PTWI=PTWI(supplied for each element)×BW

where: PTWI for Al—2 mg/kg BW; Pb—25 µg/kg BW; Cd—7 µg/kg BW; Hg—1.6 µg/kg BW [23].

### 2.6. Statistical Analyses

Statistical analyses were performed with Statistica 13.0 PL (StatSoft, Kraków, Poland). Statistical testing included determining the arithmetic means of metal concentrations with standard deviations (SD), minimum and maximum values. Significant differences in trace element content in female fish gonads, testicles, and muscles were estimated with a one-way analysis of variance as a significance level of *p* < 0.05 (ANOVA). The significance of differences among groups was tested with Tukey’s post-hoc test (*p* < 0.05). The interrelationships within and outside groups were described using Pearson’s correlation coefficients (*p* < 0.05).

## 3. Results

### 3.1. Contents of Essential Trace Elements and Toxic Metals in Freshwater Fishes

**Figure 2:**
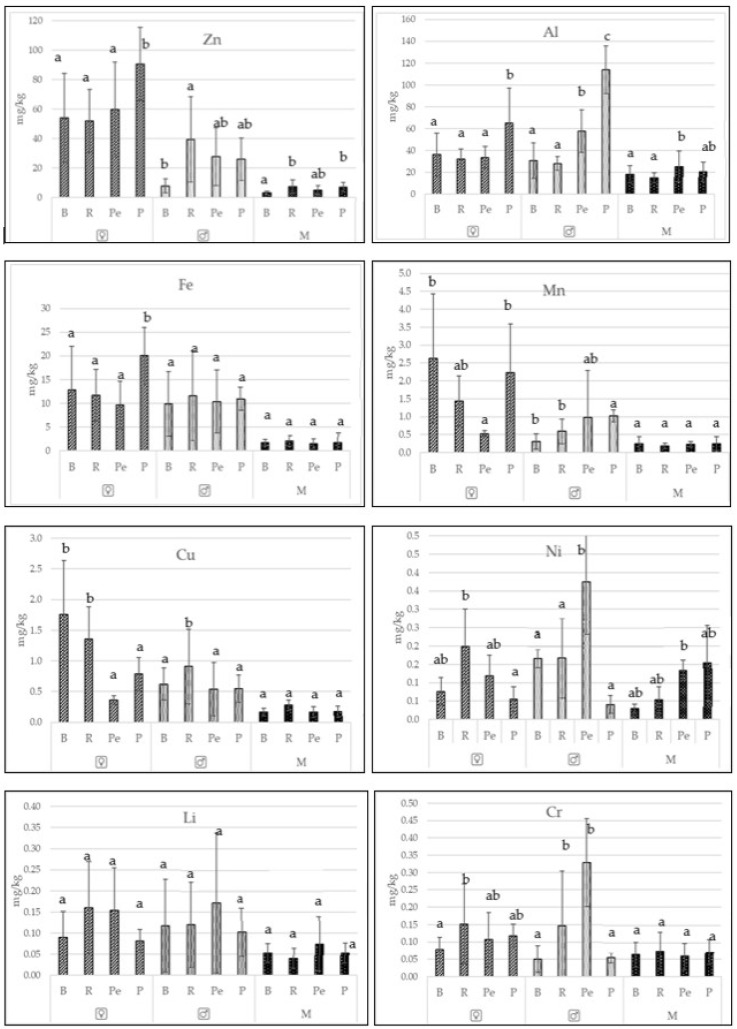
Differences (x ± SD) among species (small letters) in essential trace element contents in female gonads (♀), testicles (♂) and muscles (M) of the bream (B), roach (R), perch (Pe), and pike (P).

**Figure 3:**
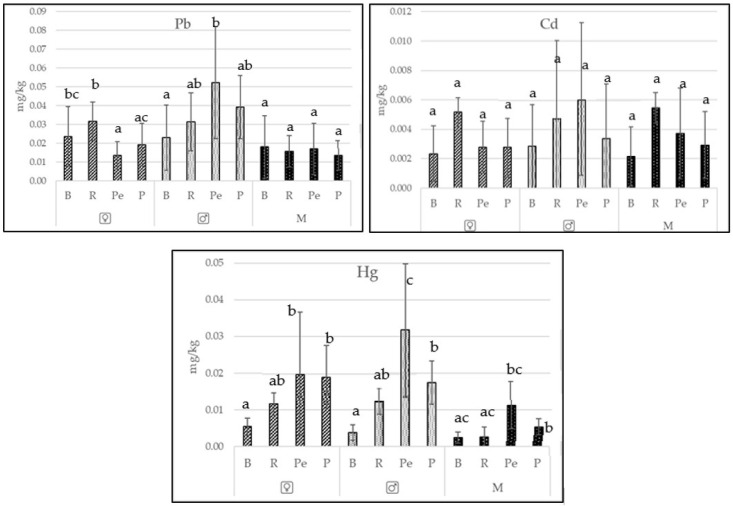
Differences (x ± SD) among species (small letters) in the content of toxic metals among female gonads (♀), testicles (♂) and muscles (M) of the bream (B), roach (R), perch (Pe), and pike (P).

The fishes collected from lakes Płoń, Miedwie, and Żelewko are among the most popular freshwater fish species caught in Poland. Table 1 presents the biometric data of the fish examined. The tested fish were characterized by very good and good condition (Table 1). The trace metal contents differed among the species. However, it was not unequivocally found that the type of feed the fish consumed was confirmed to significantly influence (*p* < 0.05) the contents of all the elements analyzed in the female gonads and testicles. Only single dependencies were observed (Figure 2 and Figure 3).

When considering the female gonads and testicles as a source of important trace elements for human nutrition, a distinction was made between female gonads and testicles (Figure 2, Table 2). Significantly higher essential trace element content was confirmed in the female gonads and testicles compared to the muscles (*p* < 0.05) (Table 2), but these differences for toxic metals were not significant statistically (*p* > 0.05) (Table 3).

In the current study, significantly higher (*p* < 0.05) zinc and iron contents were noted in the female gonads and testicles of all fishes in comparison to those in the muscles. The copper content was also significantly lower in the muscles of the fishes with the exception of perch. The content of toxic elements did not differ significantly, except in that of lead in the muscles and female gonads and testicles of pike (significant differences confirmed) (Figure 2 and Figure 3).

Metal contents can change seasonally in female gonads and testicles, which is why Table 4 presents the values of selected elements in different seasons of the year. Unfortunately, not all fish species were caught in each season, particularly pike, the abundance of which is on the decline in the lakes of West Pomerania. Table 5 presents the relationship between the content of elements in gonads and muscles in particular seasons of the year. Only in a few cases strong correlations, both negative and positive, were observed. However, no general conclusions can be drawn. This is also why the aspect of seasonality was omitted from further analysis in this study.

### 3.2. Human Health Risk

Fish consumption in Poland in 2019 was 12.7 kg per person/year (for adults) [17], which was 34.8 g/day per person. The coverage of the daily requirement for micronutrients (ADI and AI) and the amount of risk resulting from the consumption of toxic elements with a portion of fish gonads are presented in Table 6. It is worth paying attention to the high coverage of the demand for zinc and iron. When considering the risk associated with the consumption of gonads in terms of PTWI, the tested gonads should be considered safe.

The EDI of trace elements with a portion of female fish gonads and testicles or muscles is presented in Table 7. THQ values in excess of 1 indicate potentially toxic effects [25]. In the current study, both THQ and TTHQ values were <1, which indicated that female gonads and testicles consumption did not pose a toxicological risk to consumers considering the contents of trace elements and especially those that are toxic (Pb, Cd, Hg).

## 4. Discussion

The accumulation of elements in aquatic organisms is influenced by many synergistic factors, including endogenous characteristics and the physiological state of the organism, nutritional behavior, diet, geographical habitat, environmental characteristics, and the tendency of the metal to undergo biomagnification in the food chain. Female fish gonads and testicles are used in the cuisines of many countries. Salted, cured grey mullet and bluefin tuna roes are known as bottarga, which is a typical preserved product from many Mediterranean countries, while ikura, tarako, and tobiko are typical Japanese foods made from the roes of salmon, walleye pollock, and flying fish, respectively [6,26].

The highest concentrations of trace elements in female gonads and testicles were of zinc and aluminum. High zinc contents are expected in female gonads and testicles since this element participates in cell division and growth during gametogenesis [27,28]. The average zinc content range in the female gonads and testicles analyzed in the current study was 31–58 mg/kg. Moniruzzaman, M. et al. [29] reported similar results. Calza, C. [30] determined values that were twice as high in the fish species examined, and Topuz, K.O. et al. [31] also observed higher values (140.3 mg/kg). However, other authors reported much lower values of 10.3–12.4 mg/kg in the female fish gonads they analyzed. High contents of aluminum in the female gonads, testicles, and muscles of the fish examined in the current study could have been caused by the water treatment facility in the vicinity of the lakes [32]. Alum, (aluminum sulfate) is used at the facility as a coagulant to remove particles, microorganisms, and organic matter. Alum sludge is recycled back from wastes to the aquatic environment. Aluminum is of low toxicity to fishes; however, because no specific function of this element is known, it is considered unnecessary for fishes. In the analyses of female gonads and testicles in the present study, aluminum occurred in high quantities, and in pike, it was as high as 90 mg/kg. The gonads of roach and perch from Lake Baikal contained significantly lower levels of this element at 6.23 mg/kg and 6.9 mg/kg [33]. The second highest element content in female gonads and testicles was that of iron, and in the present study, the highest content exceeded 30 mg/kg in the female gonads and testicles of pike. Topuz, K.O. et al. [31] reported much higher contents in the gonads exceeding 90 mg/kg. Similarly, Niemiec, M. et al. [34] reported the male and female gonads to have high concentrations of 98.0 and 71.5 mg/kg, respectively. Manganese and copper are essential microelements, but at high concentrations, they can be extremely toxic [35]. Copper accumulates in fish gonads, and it influences, inter alia, spermatogenesis and egg hatching [36]. In the present study, the range of Mn and Cu concentrations in the female gonads and testicles oscillated around 1 mg/kg. However, some authors [37] observed much lower values for manganese (0.1 and 0.4 mg/kg). Sapozhnikova, Y. et al. [33] reported slightly higher manganese concentrations in fish from the Dniester River at 3.63 mg/kg in roach and 1.55 mg/kg in perch, while Topuz, K.O. et al. [31] reported significantly higher concentrations of both elements at 18.5 mg/kg for manganese and 18.2 mg/kg for copper. Franco-Fuentes et al. [38] reported lower concentrations of manganese at 0.1–0.4 mg/kg and higher copper content of 0.8–4.2 mg/kg. Chromium is considered to be a nonessential element in aquatic organisms that can lead to limited growth and development [39]. The highest chromium contents in the female gonads and testicles analyzed in the current study were in perch at 0.197 ± 0.350 mg/kg. Bekhit, A.D. et al. [4] confirmed lower values of this element at <0.05 mg/kg in the gonads of popular fishes in New Zealand. Perch from the Dniester River had similar quantities of this element at 0.08–0.25 mg/kg, while roach had much less of it at 0.01–0.02 mg/kg [33]. In the current study, the average levels of nickel and lithium in the female gonads and testicles of the fishes examined were 0.1–0.2 mg/kg. Unfortunately, since there was a lack of reports in the literature on nickel and lithium contents in female gonads and testicles, comparisons were only made for determinations of these elements in muscles. The Ni content in predatory fish muscles was confirmed to be more than twice that of this element in the muscles of non-predatory fishes. Lima, M.W. et al. [40] reported similar observations in fish species from the southeastern Carajás Mineral Province in Brazil. 

Biomagnification is typical for Pb, Cd, and Hg, therefore increased values of toxic elements may occur in individuals leading a predatory lifestyle (e.g., perch, pike) [41]. The scientific literature supports this assumption, also stating that Pb, Cd, and Hg concentrations are much higher than those found in our study. The results of the linear regression analysis showed a positive relationship between the concentrations of toxic metals and the length of the fish (r 0.49–0.65; *p* <0.001). Lead, cadmium, and mercury are toxic, nonessential elements [42]. Pb decreases fish survival, growth rates, and development [43]. Like lead, cadmium disrupts fish reproduction and hormone levels [44]. Analyses performed for the present study confirmed lower lead levels in fish female gonads and testicles than those reported by Wirth et al. (2000; 0.06–0.15 mg/kg) [45], Has-Schön et al. [46] (0.317 ± 0.076 mg/kg), and Anandkumar, A. et al. [47] (0.5–4.25 mg/kg ww). Similar dependencies were noted for cadmium. Some authors [38] reported a Cd content range of 0.1–0.6 mg/kg in fish gonads, while others reported a range of 0.49–1.25 mg/kg [37]. In their analysis of mercury in fish gonads, Morcillo, P. et al. [48] confirmed a significantly (*p* < 0.05) higher content than that in the present study.

### Human Health Risk

Recently, there has been increasing interest in caviar substitutes since the availability of natural caviar is limited, inter alia, by the threat of extinction of sturgeon species. Furthermore, using the roes of other fish species in products such as sushi is being proposed with increasing frequency. The estimated global market for processed fish roe is 60,000 t, while actual caviar production is under 500 t [49,50].

Health risks regarding the consumption of Pb, Cd, and Hg-contaminated fish are subject to regulation introduced by many countries and government agencies. The regulation of the European Commission [51] specifies the maximum levels for the content of these elements only in the muscles of fish. The Joint Food and Agriculture Organization of the United Nations and World Health Organization FAO/WHO Expert Committee on Food Additives (JECFA) has established provisional tolerable weekly intake (PTWI) for Pb, Cd, Hg as the amount of contaminant that can be consumed throughout life without significant risk.

The TTHQ values determined for these elements were significantly lower than 1, which meant that female gonads and testicles can be used successfully as consumer products. The results of other researchers also confirm this [52,53,54]. However, some studies do confirm THQ values above 1; for example, Lima M.W. et al. [40] confirmed THQ_Pb_ in the range of 1.75–3.60, which could pose a threat to consumers since lead can contribute to lung cancer and brain and liver damage [55]. Lima, M.W. et al. [40] also confirmed very high values of TTHQ in excess of 2.66 that they attributed to, inter alia, low water quality and lead contamination in bottom sediments. The calculated risk factors in this study, indicate a negligible probability of health risk arising from the consumption of the studied gonads, however, due to the limited amount of available literature data, it is difficult to exclude that such a risk may occur elsewhere, especially in people based on fish and fish products.

## 5. Conclusions

Despite the growing significance of fish gonad products on the international market, little information is available on their proximate composition, quality characteristics, or their content of toxic compounds. The current study analyzed the quality of female fish gonads and testicles in terms of the content in them of 11 trace elements, including toxic elements. The results of the study indicate that female gonads and testicles are valuable sources of microelements, particularly of zinc and iron, while the contents of nonessential elements do not pose threats to consumer health (TTHQ < 1). This is why it is worth considering widening the utilization of fish female gonads and testicles in food processing while also focusing on the origin of these raw materials.

## Figures and Tables

**Table 1:** Biometric measurements of the fishes examined.

Species	*n*	Fish Weight (g)		Fish Length (cm)		Gonad Weight (g)		Fulton’s Condition Factor	
		x	SD	x	SD	x	SD	x	SD
		(Min–Max)		(Min–Max)		(Min–Max)		(Min–Max)	
Bream *(Abramis brama)*	120	654.45	285.53	37.20	5.50	24.51	37.06	1.20	0.15
		(170.1–1360.6)		(25.3–49.0)		(1.4–230.7)		(0.88–1.51)	
Roach *(Rutilus rutilus)*	120	147.33	48.94	22.81	2.41	7.94	10.22	1.20	0.17
		(5.52–350.4)		(17–30)		(0.5–53.5)		(0.75–1.78)	
Perch *(Perca fluviatilis)*	120	157.47	35.48	23.18	1.67	2.67	6.97	1.25	0.13
		(94.2–240.4)		(20.1–26.5)		(0.2–40.5)		(1.05–1.62)	
Pike *(Esox lucius)*	90	2811.39	10621.40	49.47	6.22	40.54	36.07	0.72	0.09
		(211.2–60,000.4)		(31.5–72.5)		(0.2–40.5)		(0.52–0.98)	

Notes: *n*—number of specimens, SD—standard deviation, min–max—minimum–maximum values.

**Table 2:** Content of essential trace elements in female fish gonads and testicles (F + T) and muscles (M) (mg/kg).

Species		Zn		Al		Fe		Mn		Cu		Ni		Li		Cr	
		F + T	M	F + T	M	F + T	M	F + T	M	F + T	M	F + T	M	F + T	M	F + T	M
Bream *(Abramis brama)*	x	**31.0**	**1.2**	**33.38**	**7.94**	**11.39**	**0.69**	**1.47**	**0.19**	**1.19**	**0.06**	**0.121**	**0.026**	0.104	0.023	0.064	0.037
	SD	31.7	1.2	18.01	7.94	8.08	0.69	1.73	0.19	0.86	0.06	0.191	0.026	0.088	0.023	0.039	0.037
	min	2.5	0.5	5.26	2.98	0.60	0.44	0.11	0.06	0.20	0.02	0.002	0.000	0.007	0.016	0.002	0.005
	max	106.6	5.2	67.57	34.46	26.48	3.26	6.70	1.07	3.02	0.31	0.937	0.116	0.428	0.103	0.133	0.150
Roach *(Rutilus rutilus)*	x	**46.2**	**7.3**	30.14	14.94	**11.65**	**2.04**	**1.04**	**0.18**	**1.15**	**0.28**	**0.184**	**0.054**	**0.142**	**0.041**	0.149	0.076
	SD	25.7	4.6	8.26	4.61	7.48	1.12	0.70	0.08	0.60	0.09	0.193	0.052	0.161	0.038	0.135	0.054
	min	5.2	2.0	16.09	3.56	0.16	0.23	0.15	0.03	0.21	0.10	0.003	0.002	0.019	0.010	0.031	0.002
	max	113.1	28.9	48.97	23.76	27.82	6.41	3.47	0.40	2.80	0.48	0.638	0.235	0.868	0.228	0.681	0.296
Perch *(Perca fluviatilis)*	x	**46.7**	**4.9**	43.36	25.22	**9.92**	**1.45**	0.71	0.23	0.43	0.16	**0.223**	**0.134**	0.161	0.074	**0.197**	**0.059**
	SD	31.7	3.1	18.57	14.28	5.63	1.01	0.84	0.08	0.29	0.09	0.419	0.227	0.129	0.064	0.350	0.039
	min	6.1	1.1	20.86	8.55	2.47	0.25	0.25	0.11	0.19	0.03	0.008	0.010	0.046	0.012	0.001	0.009
	max	154.1	11.9	85.77	63.17	23.40	4.98	4.44	0.44	1.67	0.37	2.081	1.185	0.592	0.216	1.726	0.183
Pike *(Esox lucius)*	x	**58.2**	**7.0**	89.42	20.48	**15.49**	**1.76**	1.63	0.25	**0.67**	**0.17**	0.048	0.103	0.092	0.053	0.086	0.072
	SD	68.3	3.1	36.90	8.73	6.43	1.95	1.13	0.20	0.27	0.09	0.046	0.153	0.045	0.023	0.041	0.036
	min	11.9	3.5	19.07	7.19	7.45	0.01	0.31	0.06	0.24	0.07	0.003	0.005	0.053	0.016	0.036	0.033
	max	344.4	15.8	166.08	56.87	30.71	10.96	3.82	0.97	1.52	0.47	0.192	0.734	0.246	0.096	0.163	0.173

Notes: Bold—differences among female gonads and testicles and muscles were significant (*p* ≤ 0.05); F—female fish gonads; T—testicles; M—muscles; SD—standard deviation; min—minimum values; max—maximum values.

**Table 3:** Content of toxic elements in female fish gonads and testicles (F + T) and muscles (M) (mg/kg).

Species		Pb		Cd		Hg	
		F + T	M	F + T	M	F + T	M
Bream	x	0.023	0.017	0.003	0.002	0.005	0.001
	SD	0.016	0.017	0.002	0.002	0.002	0.001
	min	0.003	0.000	0.000	0.000	0.001	0.001
	max	0.063	0.060	0.011	0.009	0.010	0.006
Roach	x	0.032	0.016	0.005	0.005	0.012	0.003
	SD	0.013	0.008	0.009	0.010	0.003	0.003
	min	0.004	0.006	0.001	0.001	0.007	0.001
	max	0.071	0.044	0.056	0.055	0.018	0.020
Perch	x	0.029	0.017	0.004	0.004	0.025	0.011
	SD	0.027	0.014	0.004	0.003	0.027	0.006
	min	0.004	0.005	0.001	0.000	0.002	0.001
	max	0.103	0.073	0.019	0.017	0.097	0.028
Pike	x	**0.029**	**0.014**	0.003	0.003	**0.018**	**0.005**
	SD	0.017	0.008	0.003	0.002	0.007	0.002
	min	0.007	0.003	0.000	0.001	0.005	0.001
	max	0.068	0.033	0.015	0.009	0.032	0.010

Notes: Bold—differences among female gonads and testicles and muscles were significant (*p* ≤ 0.05); F—female fish gonads; T—testicles; M—muscles; SD—standard deviation; min—minimum values; max—maximum values.

**Table 4:** Content of trace elements in female gonads and testicles in different seasons of the year.

	Spring				Summer				Autumn				Winter			
	Female Gonads		Testicles		Female Gonads		Testicles		Female Gonads		Testicles		Female Gonads		Testicles	
	x	SD	x	SD	x	SD	x	SD	x	SD	x	SD	x	SD	x	SD
	ZINC mg/kg															
Bream	28.92 ^A^	9.62	6.73 ^W^	3.18	99.42 ^B^	9.14	7.07 ^W^	2.53	69.18 ^B^	17.08	9.92 ^W^	7.00	n.s.	n.s.	n.s.	n.s.
Roach	48.75 ^A^	18.09	19.26 ^W^	11.85	70.77 ^A^	40.26	37.98 ^Y^	23.20	n.s.	n.s.	n.s.	n.s.	n.s.	n.s.	n.s.	n.s.
Perch	36.52 ^A^	12.29	42.61 ^W^	26.97	43.30 ^A^	15.22	58.43 ^Y^	27.81	88.07 ^B^	8.23	n.s.	n.s.	65.11 ^B^	10.73	12.85 ^Y^	6.33
Pike	97.16 ^A^	18.03	40.53 ^W^	11.26	215.21 ^B^	132.86	n.s.	n.s.	46.98 ^C^	12.33	16.01 ^Y^	3.51	n.s.	n.s.	n.s.	n.s.
MEAN	44.07	24.26	26.21	22.04	86.34	75.31	41.34	29.80	61.23	20.55	13.57	5.84	65.11	10.73	12.85	6.33
	ALUMINUM mg/kg															
Bream	25.1 ^B^	12.8	31.8 ^Y^	11.6	49.0 ^A^	3.9	23.1 ^Y^	1.3	46.2 ^A^	24.8	32.9 ^Y^	25.2	n.s.	n.s.	n.s.	n.s.
Roach	32.2 ^A^	9.2	57.8 ^Y^	24.1	35.1 ^A^	10.9	57.2 ^Y^	14.9	n.s.	n.s.	n.s.	n.s.	n.s.	n.s.	n.s.	n.s.
Perch	33.2 ^A^	11.8	28.5 ^Y^	6.5	31.4 ^A^	9.2	29.1 ^Y^	7.1	29.9 ^A^	4.8	n.s.	n.s.	31.9 ^A^	10.1	26.3	5.5
Pike	25.1 ^B^	6.3	106.2	18.6	77.1 ^A^	55.1	n.s.	n.s.	74.1 ^A^	18.1	119.1	23.3	n.s.	n.s.	n.s.	n.s.
MEAN	29.42	11.17	51.63	33.78	41.84	24.95	37.28	17.43	57.40	25.76	84.65	49.49	31.89	10.06	26.31	5.45
	IRON mg/kg															
Bream	5.5 ^B^	4.1	6.1 ^W^	6.1	24.8 ^A^	1.5	17.8 ^Y^	7.3	18.0 ^B^	6.4	11.6 ^Y^	2.9	n.s.	n.s.	n.s.	n.s.
Roach	11.9 ^A^	5.6	11.5 ^W^	8.1	7.4 ^A^	3.5	9.1 ^W^	4.9	n.s.	n.s.	n.s.	n.s.	n.s.	n.s.	n.s.	n.s.
Perch	14.1 ^A^	7.4	16.4 ^W^	8.9	11.4 ^A^	4.5	14.3 ^W^	8.5	14.1 ^A^	1.4	n.s.	n.s.	7.7 ^A^	1.5	2.1 ^Y^	1.1
Pike	18.7 ^A^	8.1	11.3 ^W^	2.6	16.2 ^A^	4.1	n.s.	n.s.	21.8 ^A^	5.7	10.8 ^W^	2.4	n.s.	n.s.	n.s.	n.s.
MEAN	11.18	7.17	11.13	7.71	12.46	6.95	13.23	7.55	19.23	6.02	11.09	2.59	7.71	1.51	2.05	1.12
	MANGANESE mg/kg															
Bream	2.1 ^A^	1.1	0.3 ^W^	0.1	0.9 ^A^	0.01	0.2 ^W^	0.06	4.4 ^B^	1.7	0.4 ^W^	0.3	n.s.	n.s.	n.s.	n.s.
Roach	0.6 ^A^	0.07	1.1 ^W^	1.6	0.5 ^A^	0.08	0.9 ^W^	0.9	n.s.	n.s.	n.s.	n.s.	n.s.	n.s.	n.s.	n.s.
Perch	1.2 ^A^	0.4	0.6 ^W^	0.3	1.2 ^A^	0.5	0.9 ^W^	0.3	1.9 ^A^	0.7	n.s.	n.s.	1.9 ^A^	0.9	0.2 ^W^	0.06
Pike	0.4 ^A^	0.07	1.1 ^W^	0.2	1.1 ^A^	0.7	n.s.	n.s.	3.22 ^B^	0.5	1.0 ^W^	0.2	n.s.	n.s.	n.s.	n.s.
MEAN	1.19	0.92	0.71	0.80	0.86	0.47	0.72	0.59	3.37	1.35	0.76	0.36	1.91	0.95	0.23	0.06
	COPPER mg/kg															
Bream	1.14 ^A^	0.68	0.49 ^W^	0.17	2.39 ^B^	0.71	0.73 ^W^	0.15	2.38 ^B^	0.51	0.76 ^W^	0.35	n.s.	n.s.	n.s.	n.s.
Roach	0.39 ^A^	0.06	0.65 ^W^	0.52	0.33 ^A^	0.07	0.41 ^W^	0.33	n.s.	n.s.	n.s.	n.s.	n.s.	n.s.	n.s.	n.s.
Perch	1.22 ^A^	0.43	0.84 ^W^	0.39	1.32 ^A^	0.79	1.37 ^W^	0.77	1.17 ^A^	0.04	n.s.	n.s.	1.67^A^	0.29	0.47 ^Y^	0.18
Pike	0.53 ^A^	0.11	0.69 ^W^	0.20	0.97 ^A^	0.53	n.s.	n.s.	0.81 ^A^	0.13	0.46 ^W^	0.19	n.s.	n.s.	n.s.	n.s.
MEAN	0.88	0.57	0.66	0.34	1.05	0.88	0.92	0.70	1.39	0.79	0.58	0.30	1.67	0.29	0.47	0.18
	NICKEL mg/kg															
Bream	0.063 ^A^	0.053	0.075 ^Y^	0.048	0.041 ^A^	0.003	0.011 ^Y^	0.014	0.113 ^A^	0.115	0.380 ^Y^	0.365	n.s.	n.s.	n.s.	n.s.
Roach	0.108 ^A^	0.062	0.476 ^Y^	0.794	0.129 ^A^	0.052	0.252 ^Y^	0.449	n.s.	n.s.	n.s.	n.s.	n.s.	n.s.	n.s.	n.s.
Perch	0.412 ^B^	0.142	0.319 ^Y^	0.208	0.173 ^A^	0.164	0.119 ^Y^	0.104	0.025 ^A^	0.02	n.s.	n.s.	0.03 ^A^	0.031	0.02 ^Y^	0.006
Pike	0.054 ^A^	0.041	0.074 ^Y^	0.064	0.075 ^A^	0.052	n.s.	n.s.	0.048 ^A^	0.045	0.018 ^Y^	0.008	n.s.	n.s.	n.s.	n.s.
MEAN	0.175	0.176	0.225	0.391	0.124	0.107	0.142	0.265	0.066	0.078	0.163	0.285	0.032	0.031	0.021	0.006
	LITHIUM mg/kg															
Bream	0.11 ^A^	0.05	0.17 ^W^	0.12	0.10 ^A^	0.08	n.s.	n.s.	0.05 ^A^	0.05	0.04 ^W^	0.02	n.s.	n.s.	n.s.	n.s.
Roach	0.17 ^A^	0.13	0.19 ^W^	0.20	0.14 ^A^	0.06	0.14 ^W^	0.13	n.s.	n.s.	n.s.	n.s.	n.s.	n.s.	n.s.	n.s.
Perch	0.33 ^B^	0.25	0.21 ^W^	0.14	0.12 ^A^	0.09	0.07 ^Y^	0.06	0.06 ^A^	0.02	n.s.	n.s.	0.03 ^A^	0.01	0.06 ^Y^	0.02
Pike	0.10 ^A^	0.04	0.08 ^W^	0.03	0.10 ^A^	0.04	n.s.	n.s.	0.07 ^A^	0.01	0.12 ^W^	0.07	n.s.	n.s.	n.s.	n.s.
MEAN	0.190	0.174	0.167	0.135	0.120	0.072	0.103	0.098	0.062	0.031	0.085	0.066	0.035	0.014	0.056	0.022
	CHROMIUM mg/kg															
Bream	0.07 ^A^	0.04	0.07 ^Y^	0.04	0.10 ^A^	0.02	0.01 ^Y^	0.01	0.08 ^A^	0.03	0.04 ^Y^	0.02	n.s.	n.s.	n.s.	n.s.
Roach	0.09 ^A^	0.03	0.41 ^Y^	0.65	0.12 ^A^	0.11	0.23 ^Y^	0.37	n.s.	n.s.	n.s.	n.s.	n.s.	n.s.	n.s.	n.s.
Perch	0.19 ^A^	0.14	0.17 ^Y^	0.14	0.18 ^A^	0.14	0.18 ^Y^	0.23	0.09 ^A^	0.01	n.s.	n.s.	0.10 ^A^	0.01	0.08 ^Y^	0.02
Pike	0.08 ^A^	0.01	0.06 ^Y^	0.01	0.12 ^A^	0.05	n.s.	n.s.	0.13 ^A^	0.02	0.05 ^Y^	0.01	n.s.	n.s.	n.s.	n.s.
MEAN	0.110	0.093	0.164	0.316	0.138	0.106	0.164	0.263	0.108	0.034	0.048	0.018	0.099	0.012	0.080	0.020
	LEAD mg/kg															
Bream	0.03 ^A^	0.02	0.03 ^Y^	0.02	0.01 ^A^	0.01	0.01 ^Y^	0.01	0.02 ^A^	0.01	0.03 ^Y^	0.02	n.s.	n.s.	n.s.	n.s.
Roach	0.01 ^A^	0.01	0.07 ^Y^	0.02	0.02 ^A^	0.01	0.03 ^W^	0.02	n.s.	n.s.	n.s.	n.s.	n.s.	n.s.	n.s.	n.s.
Perch	0.03 ^A^	0.01	0.03 ^Y^	0.02	0.04 ^A^	0.01	0.04 ^Y^	0.01	0.03 ^A^	0.01 ^Y^	n.s.	n.s.	0.03 ^A^	0.00	0.02 ^Y^	0.00
Pike	0.01 ^A^	0.01	0.04	0.02	0.01 ^A^	0.01	n.s.	n.s.	0.02 ^A^	0.01	0.04	0.02	n.s.	n.s.	n.s.	n.s.
MEAN	0.022	0.015	0.040	0.024	0.021	0.013	0.031	0.019	0.024	0.012	0.034	0.019	0.031	0.004	0.018	0.003
	CADMIUM mg/kg *															
Bream	0.002	0.001	0.002	0.003	0.001	0.000	0.001	0.000	0.004	0.002	0.004	0.003	n.s.	n.s.	n.s.	n.s.
Roach	0.003	0.002	0.006	0.006	0.003	0.002	0.007	0.004	n.s.	n.s.	n.s.	n.s.	n.s.	n.s.	n.s.	n.s.
Perch	0.002	0.001	0.004	0.003	0.011	0.020	0.008	0.008	0.002	0.000	n.s.	n.s.	0.004	0.001	0.002	0.000
Pike	0.003	0.001	0.003	0.001	0.003	0.003	n.s.	n.s.	0.002	0.002	0.004	0.005	n.s.	n.s.	n.s.	n.s.
MEAN	0.002	0.001	0.004	0.004	0.005	0.012	0.006	0.006	0.003	0.002	0.004	0.004	0.004	0.001	0.002	0.000
	MERCURY mg/kg *															
Bream	0.005	0.002	0.004	0.002	0.007	0.004	0.004	0.002	0.005	0.002	0.004	0.003	n.s.	n.s.	n.s.	n.s.
Roach	0.018	0.023	0.031	0.031	0.021	0.032	0.033	0.028	n.s.	n.s.	n.s.	n.s.	n.s.	n.s.	n.s.	n.s.
Perch	0.013	0.003	0.013	0.003	0.010	0.002	0.011	0.004	0.011	0.004	n.s.	n.s.	0.012	0.004	0.012	0.003
Pike	0.028	0.003	0.018	0.005	0.015	0.003	n.s.	n.s.	0.018	0.009	0.017	0.007	n.s.	n.s.	n.s.	n.s.
MEAN	0.014	0.014	0.015	0.016	0.015	0.020	0.017	0.020	0.012	0.009	0.012	0.009	0.012	0.004	0.012	0.003

Notes: n.s.—no samples were taken. Uppercase: ^A,B,C^—significant differences (*p* < 0.05) in the concentration of metals in the female gonads of the selected fish species depending on the season (in line). Uppercase: ^W,Y^—significant differences (*p* < 0.05) in the concentration of metals in the nucleus of a given fish species depending on the season (in line); *—in the case of Cd and Hg—no significant differences were found between sampling seasons.

**Table 5:** Pearson’s correlation coefficients (r) between the content of minerals in fish gonads and muscles in the studied seasons (*p* < 0.05).

	Species	SPRING		SUMMER		AUTUMN		WINTER	
		F v Mf	T v Mt	F v Mf	T v Mt	F v Mf	T v Mt	F v Mf	T v Mt
Zn	Bream	0.276	−0.021	−0.530	−0.809	**−0.836**	−0.504	n.s.	n.s.
	Roach	0.156	0.404	−0.220	−0.560	0.250	n.s.	−0.387	0.489
	Perch	**−0.907**	−0.632	0.432	−0.334	n.s.	n.s.	n.s.	n.s.
	Pike	−0.343	−0.436	**−0.952**	n.s.	0.183	0.618	n.s.	n.s.
Al	Bream	0.659	−0.200	0.223	−0.592	**−0.886**	−0.175	n.s.	n.s.
	Roach	0.149	−0.178	0.348	0.472	−0.296	n.s.	**−0.821**	0.625
	Perch	−0.546	−0.686	0.477	−0.361	n.s.	n.s.	n.s.	n.s.
	Pike	**−0.916**	0.007	**0.970**	n.s.	0.699	−0.595	n.s.	n.s.
Fe	Bream	−0.554	−0.525	0.139	−0.518	0.385	−0.745	n.s.	n.s.
	Roach	0.485	0.662	0.445	0.254	**0.992**	n.s.	−0.335	0.705
	Perch	−0.368	**0.911**	−0.477	−0.052	n.s.	n.s.	n.s.	n.s.
	Pike	0.076	0.575	**0.966**	n.s.	−0.110	−0.516	n.s.	n.s.
Mn	Bream	−0.534	−0.309	−0.262	**−0.826**	−0.314	**−0.997**	n.s.	n.s.
	Roach	0.176	0.32	0.565	−0.154	**−0.906**	n.s.	−0.204	−0.227
	Perch	0.561	0.595	0.617	0.074	n.s.	n.s.	n.s.	n.s.
	Pike	0.422	0.043	0.114	n.s.	0.052	**0.855**	n.s.	n.s.
Cu	Bream	−0.719	−0.190	0.757	0.127	−0.116	**−0.895**	n.s.	n.s.
	Roach	−0.010	0.390	−0.777	−0.515	**−0.925**	n.s.	−0.543	0.317
	Perch	**−0.963**	0.376	**0.948**	−0.448	n.s.	n.s.	n.s.	n.s.
	Pike	**0.940**	0.487	0.634	n.s.	0.633	−0.419	n.s.	n.s.
Ni	Bream	−0.514	−0.393	**0.877**	**0.815**	**−0.995**	**0.998**	n.s.	n.s.
	Roach	0.263	0.208	−0.509	−0.165	−0.513	n.s.	**0.813**	−0.328
	Perch	0.593	−0.015	−0.429	−0.211	n.s.	n.s.	n.s.	n.s.
	Pike	0.217	−0.313	0.779	n.s.	**0.902**	0.219	n.s.	n.s.
Li	Bream	0.739	−0.342	0.593	−0.377	−0.805	**−0.944**	n.s.	n.s.
	Roach	0.229	0.124	−0.163	0.332	0.513	n.s.	0.769	**0.965**
	Perch	−0.644	0.598	−0.451	0.578	n.s.	n.s.	n.s.	n.s.
	Pike	−0.615	0.169	−0.627	n.s.	−0.013	−0.121	n.s.	n.s.
Cr	Bream	**−0.870**	0.299	0.314	0.683	0.494	0.734	n.s.	n.s.
	Roach	**0.891**	0.152	**0.983**	0.229	−0.324	n.s.	−0.044	**−0.849**
	Perch	−0.679	0.647	0.634	0.618	n.s.	n.s.	n.s.	n.s.
	Pike	0.145	0.249	**−0.970**	n.s.	0.277	0.418	n.s.	n.s.
Pb	Bream	−0.104	−0.249	−0.251	0.671	**0.964**	**0.977**	n.s.	n.s.
	Roach	0.407	0.574	**−0.891**	−0.670	0.541	n.s.	0.418	−0.502
	Perch	0.231	−0.060	0.611	−0.071	n.s.	n.s.	n.s.	n.s.
	Pike	−0.097	0.121	0.731	n.s.	0.657	−0.788	n.s.	n.s.
Cd	Bream	0.462	−0.055	−0.452	−0.198	**−0.851**	**0.991**	n.s.	n.s.
	Roach	0.050	0.247	0.517	−0.176	0.733	n.s.	−0.506	−0.482
	Perch	0.573	−0.061	−0.118	0.486	n.s.	n.s.	n.s.	n.s.
	Pike	**0.985**	−0.503	**−0.873**	n.s.	−0.205	−0.319	n.s.	n.s.
Hg	Bream	−0.385	−0.414	0.174	-0.493	−0.839	−0.277	n.s.	n.s.
	Roach	0.176	−0.09	−0.724	−0.452	−0.958	n.s.	0.469	−0.603
	Perch	0.163	0.239	0.667	−0.498	n.s.	n.s.	n.s.	n.s.
	Pike	−0.549	−0.805	0.484	n.s.	−0.528	0.247	n.s.	n.s.

Notes: F—female fish gonads; T—testicles; Mf—female muscles; Mt—male muscles; n.s.—no samples were taken; bold—strong correlation (*p* < 0.05).

**Table 6:** Coverage of the daily requirement for micronutrients (%) and the degree of risk resulting from the intake of toxic elements with a portion of fish gonads (% PTWI).

		Recommended Dietary Allowance %												Adequate Intake%				Provisional Tolerable Weekly Intake %							
		Zn				Fe				Cu		Li		Mn				Al		Pb		Cd		Hg	
		Women		Men		Women		Men		Adult		Adult		Women		Men									
		x	SD	x	SD	x	SD	x	SD	x	SD	x	SD	x	SD	x	SD	x	SD	x	SD	x	SD	x	SD
Bream	F	24	13	17	10	5.6	4.0	4.1	2.9	6.8	3.4	0.3	0.2	5.1	3.5	4.0	2.7	0.9	0.5	0.05	0.03	0.02	0.01	0.07	0.03
	T	3	2	2	1	4.3	3.0	3.1	2.1	2.4	1.0	0.4	0.4	0.6	0.4	0.5	0.3	0.8	0.4	0.05	0.03	0.02	0.02	0.05	0.03
Perch	F	26	14	19	10	4.2	2.2	3.0	1.6	1.4	0.3	0.5	0.4	1.0	0.2	0.8	0.1	0.8	0.2	0.03	0.01	0.02	0.01	0.25	0.33
	T	12	8	9	6	4.5	2.9	3.3	2.1	2.1	1.7	0.6	0.6	1.9	2.5	1.5	2.0	1.4	0.5	0.10	0.06	0.04	0.04	0.05	0.02
Roach	F	23	9	16	7	5.1	2.4	3.7	1.7	5.2	2.0	0.6	0.7	2.8	1.4	2.2	1.1	0.8	0.2	0.06	0.02	0.04	0.08	0.14	0.04
	T	17	13	12	9	5.0	4.1	3.7	3.0	3.5	2.4	0.4	0.4	1.1	0.7	0.9	0.5	0.7	0.2	0.06	0.03	0.03	0.04	0.15	0.04
Pike	F	39	37	29	27	8.7	2.6	6.3	1.9	3.0	1.1	0.3	0.1	4.3	2.6	3.4	2.1	1.6	0.8	0.04	0.02	0.02	0.01	0.24	0.11
	T	11	6	8	5	4.8	1.1	3.5	0.8	2.1	0.9	0.4	0.2	2.0	0.3	1.5	0.3	2.8	0.5	0.08	0.03	0.02	0.03	0.22	0.07

Notes: F—female fish gonads; T—testicles; SD—standard deviation.

**Table 7:** Estimated daily intake of elements with a portion of fish (EDI, THQ, TTHQ).

Species			Zn	Ni	Fe	Mn	Cr	Cu	Al	Li	Pb	Cd	Hg
			Estimated Daily Intake (EDI)										
Bream	F	x	26.89	0.038	6.404	1.304	0.039	0.875	17.93	0.045	0.012	0.001	0.003
		SD	15.06	0.038	4.546	0.896	0.018	0.434	9.82	0.030	0.008	0.001	0.001
	T	x	3.90	0.082	4.922	0.153	0.025	0.309	15.26	0.058	0.011	0.001	0.002
		SD	2.34	0.127	3.374	0.107	0.019	0.130	8.05	0.054	0.009	0.001	0.001
	M	x	1.55	0.015	0.837	0.125	0.032	0.082	9.03	0.026	0.009	0.001	0.001
		SD	0.59	0.013	0.341	0.095	0.018	0.032	3.95	0.011	0.008	0.001	0.001
Roach	F	x	25.90	0.099	5.820	0.711	0.075	0.675	15.88	0.080	0.016	0.003	0.006
		SD	10.64	0.100	2.696	0.348	0.057	0.259	4.70	0.096	0.005	0.005	0.002
	T	x	19.58	0.083	5.757	0.295	0.073	0.453	13.95	0.060	0.016	0.002	0.006
		SD	14.40	0.093	4.695	0.170	0.078	0.303	3.10	0.058	0.008	0.003	0.002
	M	x	3.61	0.027	1.012	0.090	0.038	0.138	7.43	0.020	0.008	0.003	0.001
		SD	2.29	0.026	0.557	0.040	0.027	0.043	2.29	0.019	0.004	0.005	0.001
Perch	F	x	29.71	0.059	4.779	0.261	0.053	0.179	16.72	0.076	0.007	0.001	0.010
		SD	16.02	0.028	2.516	0.040	0.039	0.035	4.90	0.050	0.004	0.001	0.013
	T	x	19.58	0.083	5.757	0.295	0.073	0.453	13.95	0.060	0.016	0.002	0.006
		SD	14.40	0.093	4.695	0.170	0.078	0.303	3.10	0.058	0.008	0.003	0.002
	M	x	2.42	0.067	0.719	0.114	0.029	0.082	12.54	0.037	0.008	0.002	0.006
		SD	1.52	0.113	0.503	0.040	0.019	0.043	7.10	0.032	0.007	0.002	0.003
Pike	F	x	45.07	0.027	9.962	1.108	0.059	0.390	32.25	0.040	0.010	0.001	0.009
		SD	42.23	0.022	2.966	0.676	0.016	0.135	16.13	0.014	0.006	0.001	0.004
	T	x	12.83	0.020	5.443	0.508	0.027	0.273	56.66	0.051	0.020	0.002	0.009
		SD	7.15	0.024	1.204	0.085	0.007	0.110	10.86	0.028	0.008	0.002	0.003
	M	x	3.48	0.051	0.875	0.123	0.036	0.086	10.18	0.026	0.007	0.001	0.003
		SD	1.53	0.076	0.968	0.101	0.018	0.043	4.34	0.012	0.004	0.001	0.001
			**Target Hazard Quotient (THQ)**										
Bream	F	x	0.090	0.002	0.009	0.009	0.013	0.022	0.045	0.002	0.003	0.001	0.027
		SD	0.050	0.002	0.006	0.006	0.006	0.011	0.025	0.002	0.002	0.001	0.012
	T	x	0.013	0.004	0.007	0.001	0.008	0.008	0.038	0.003	0.003	0.001	0.019
		SD	0.008	0.006	0.005	0.001	0.006	0.003	0.020	0.003	0.002	0.001	0.011
	M	x	0.005	0.001	0.001	0.001	0.011	0.002	0.023	0.001	0.003	0.001	0.012
		SD	0.002	0.001	0.000	0.001	0.006	0.001	0.010	0.001	0.002	0.001	0.007
Roach	F	x	0.086	0.005	0.008	0.005	0.025	0.017	0.040	0.004	0.004	0.003	0.058
		SD	0.035	0.005	0.004	0.002	0.019	0.006	0.012	0.005	0.001	0.005	0.016
	T	x	0.065	0.004	0.008	0.002	0.024	0.011	0.035	0.003	0.004	0.002	0.062
		SD	0.048	0.005	0.007	0.001	0.026	0.008	0.008	0.003	0.002	0.003	0.018
	M	x	0.012	0.001	0.001	0.001	0.013	0.003	0.019	0.001	0.002	0.003	0.013
		SD	0.008	0.001	0.001	0.000	0.009	0.001	0.006	0.001	0.001	0.005	0.014
Perch	F	x	0.099	0.003	0.007	0.002	0.018	0.004	0.042	0.004	0.002	0.001	0.099
		SD	0.053	0.001	0.004	0.000	0.013	0.001	0.012	0.003	0.001	0.001	0.133
	T	x	0.065	0.004	0.008	0.002	0.024	0.011	0.035	0.003	0.004	0.002	0.062
		SD	0.048	0.005	0.007	0.001	0.026	0.008	0.008	0.003	0.002	0.003	0.018
	M	x	0.008	0.003	0.001	0.001	0.010	0.002	0.031	0.002	0.002	0.002	0.056
		SD	0.005	0.006	0.001	0.000	0.006	0.001	0.018	0.002	0.002	0.002	0.032
Pike	F	x	0.150	0.001	0.014	0.008	0.020	0.010	0.081	0.002	0.003	0.001	0.094
		SD	0.141	0.001	0.004	0.005	0.005	0.003	0.040	0.001	0.002	0.001	0.043
	T	x	0.043	0.001	0.008	0.004	0.009	0.007	0.142	0.003	0.006	0.002	0.086
		SD	0.024	0.001	0.002	0.001	0.002	0.003	0.027	0.001	0.002	0.002	0.029
	M	x	0.012	0.003	0.001	0.001	0.012	0.002	0.025	0.001	0.002	0.001	0.027
		SD	0.005	0.004	0.001	0.001	0.006	0.001	0.011	0.001	0.001	0.001	0.011
			**Total Target Hazard Quotient (TTHQ)**										
Bream	F							0.223					
	T							0.106					
	M							0.060					
Roach	F							0.255					
	T							0.222					
	M							0.069					
Perch	F							0.280					
	T							0.222					
	M							0.119					
Pike	F							0.384					
	T							0.309					
	M							0.087					

Notes: F—female fish gonads; T—testicles; M-muscles; SD—standard deviation.

## Data Availability

Not applicable.

## References

[1] Maleki A., Azadi N.A., Mansouri B., Majnoni F., Rezaei Z., Gharibi F. (2015). Health risk assessment of trace elements in two fish species of Sanandaj Gheshlagh Reservoir, Iran. Toxicol. Environ. Health Sci..

[2] Varol M., Kaya G.K., Alp S.A., Sünbül M.R. (2018). Trace metal levels in rainbow trout (*Oncorhynchus mykiss*) cultured in net cages in a reservoir and evaluation of human health risks from consumption. Biol. Trace Elem. Res..

[3] Ravanbakhsh M., Javid A.Z., Hadi M., Fard N.J.H. (2020). Heavy metals risk assessment in fish species (*Johnius Belangerii* (C) and *Cynoglossus Arel*) in Musa Estuary, Persian Gulf. Environ. Res..

[4] Bekhit A.-D., Morton J.D., Dawson C.O. (2008). Effect of processing conditions on trace elements in fish roe from six commercial New Zealand fish species. J. Agric. Food Chem..

[5] Farag M.A., Abib B., Tawfik S., Shafik N., Khattab A.R. (2021). Caviar and fish roe substitutes: Current status of their nutritive value, bio-chemical diversity, authenticity and quality control methods with future perspectives. Trends Food Sci. Tech..

[6] Vasconi M., Tirloni E., Stella S., Coppola C., Lopez A., Bellagamba F., Bernardi C., Moretti V.M. (2020). Comparison of chemical composition and safety issues in fish roe products: Application of chemometrics to chemical data. Foods.

[7] Jiang H., Qin D., Chen Z., Tang S., Bai S., Mou Z. (2016). Heavy metal levels in fish from Heilongjiang River and potential health risk assessment. Bull. Environ. Contam. Toxicol..

[8] Griboff J., Wunderlin D.A., Monferran M.V. (2017). Metals, As and Se determination by inductively coupled plasma-mass spectrometry (ICP-MS) in edible fish collected from three eutrophic reservoirs their consumption represents a risk for human health?. Microchem. J..

[9] US Environmental Protection Agency (USEPA) (2000). Guidance for Assessing Chemical Contaminant Data for Use in Fish Advisories, Volume II. Risk Assessment and Fish Consumption Limits.

[10] Varol M., Sünbül M.R. (2017). Comparison of heavy metal levels of farmed and escaped farmed rainbow trout and health risk assessment associated with their consumption. Environ. Sci. Pollut. Res..

[11] Daniszewski P., Konieczny R. (2013). Heavy Metal Content in Water of Miedwie Lake (North-West Poland). Int. Lett. Chem. Phys. Astron..

[12] Bojakowska G., Sokołowska G. (1998). Geochemical classes of water sediment purity. Przeg. Geolog..

[13] MacDonald D.D., Ingersoll C.G., Berger T.A. (2000). Development and Evaluation of Consensus-Based Sediment Quality Guidelines for Freshwater Systems. Arch. Environ. Contam. Toxicol..

[14] Bakierowska A., Bursztynowicz M., Bykowszczenko N., Chałupińska J., Gajdecki A., Jurkowska K., Kordas A., Landsberg-Uczciwek M., Michalska M., Miluch A. (2018). The State of the Environment in the West Pomeranian Voivodeship 2018 Report. Voivodship Inspectorate of Environmental Protection in Szczecin. https://wios.szczecin.pl/chapter_16003.asp.

[15] Czerniejewski P., Czerniawski R. (2016). Ryby Słodkowodne i Morskie Polski [Freshwater and Marine Fishes of Poland].

[16] Froese R. (2006). Cube law, condition factor and weight–length relationships: History, meta-analysis and recommendations. J. Appl. Ihthyol..

[17] (2020). Statistical Yearbook of Agriculture. https://stat.gov.pl/en/topics/statistical-yearbooks/statistical-yearbooks/statistical-yearbook-of-agriculture-2020,6,15.html.

[18] Jarosz M. (2017). Human Nutrition Recommendations for Polish Population.

[19] Copat C., Arena G., Fiore M., Ledda C., Fallico R., Sciacca S., Ferrante M. (2013). Heavy metals concentrations in fish and shellfish from eastern Mediterranean Sea: Consumption advisories. Food Chem. Toxicol..

[20] (1989). Risk Assessment Guidance for Superfund. Volume I: Human Health Evaluation Manual (Part A).

[21] USEPA (2011). USEPA Regional Screening Level (RSL) Summary Table: November 2011. (HI).

[22] Copat C., Conti G.O., Fallico R., Sciacca S., Ferrante M., Preedy V.R., Watson R.R. (2015). Chapter 49—Heavy metals in fish from the Mediterranean Sea: Potential impact on diet. The Mediterranean Diet.

[23] European Food Safety Authority (EFSA) (2012). Scientific Opinion on the risk for public health related to the presence of mercury and methylmercury in food. EFSA.

[24] Ulusoy S., Mol S. (2020). Mercury intake via consumption of imported Atlantic mackerel (*Scomber scombrus*) in Istanbul. Int. J. Agric. Food Sci..

[25] USEPA (2001). Supplemental Guidance for Developing Soil Screening Levels for Superfund Sites.

[26] Shirai N., Higuchi T., Suzuki H. (2006). Analysis of lipid classes and the fatty acid composition of the salted fish roe food products, Ikura, Tarako, Tobiko and Kazunoko. Food Chem..

[27] Lima R.G., Araújo F.G., Maia M.F., da Silveira Braz Pinto A.S. (2002). Evaluation of heavy metals in fish of Sepetiba and Ilha Grande Bays, Rio de Janeiro, Brazil. Environ. Res..

[28] Olmedo P., Hernández A.F., Pla A., Femia P., Navas-Acien A., Gil F. (2013). Determination of essential elements (copper, manganese, selenium and zinc) in fish and shellfish samples. Risk and nutritional assessment and mercury-selenium balance. Food Chem. Toxicol..

[29] Moniruzzaman M., Das D., Dhara A., Chakraborty S.B. (2020). Enzymatic, non-enzymatatic antioxidant levels and heat shock protein expression as indicators of metal induced toxicity and reproductive modulation in female indian major carp Cirrhinus cirrhosus. Bull. Environ. Contam. Toxicol..

[30] Calza C., Anjos M.J., Castro C.R., Barroso R., Araújo F.G., Lopes R.T. (2004). Evaluation of heavy metals levels in the Paraíba do Sul River by SRTXRF in muscle, gonads and gills of Geophagus brasiliensis. Radiat. Phys. Chem..

[31] Topuz K.O., Yerlikaya P., Yatmaz H.A., Kaya A., Alp A.C., Kiliç M. (2017). Comparison of essential trace element profiles of rainbow trout fish (Oncorhynchus mykiss) meat and egg. Anim. Sci. Sci. Pap. Ser. D.

[32] Mortula M., Bard S.M., Walsh M.E., Gagnon G.A. (2008). Aluminum toxicity and ecological risk assessment of dried alum residual into surface water disposal. Can. J. Civ. Eng..

[33] Sapozhnikova Y., Zubcov N., Hungerford S., Roy L.A., Boicenco N., Zubcov E., Schlenk D. (2005). Evaluation of pesticides and metals in fish of the Dniester River, Moldova. Chemosphere.

[34] Niemiec M., Kuboń M., Komorowska M., Kuzminova N., Sikora J., Szeląg-Sikora A. (2019). Content of Cd, Cu, Cr, Fe Mn, Ni and Pb in water and selected organs of blotched Picarel Spicara maena and Mezgit Merlangius euxmus L. from Karantinna Bay and Balaklava Bay in the region of Sevastopol Middle Pomeranian. Annu. Set Environ. Prot..

[35] Gárriz Á., Del Fresno P.S., Carriquiriborde P., Miranda L.A. (2019). Effects of heavy metals identified in Chascomús shallow lake on the endocrine-reproductive axis of pejerrey fish (Odontesthes bonariensis). Gen. Comp. Endocrinol..

[36] Lin S., Taylor A.A., Ji Z., Chang C.H., Kinsinger N.M., Ueng W., Walker S.L., Nel A.E., Zhaoxia J. (2015). Understanding the transformation, speciation, and hazard potential of copper particles in a model septic tank system using zebrafish to monitor the effluent. ACS Nano.

[37] Türkmen M., Türkmen A., Tepe Y. (2011). Comparison of metals in tissues of fish from paradeniz lagoon in the coastal area of northern East Mediterranean. Bull. Environ. Contam. Toxicol..

[38] Franco-Fuentes E., Moity N., Ramírez-González J., Andrade-Vera S., Hardisson A., Gonzalez-Weller D., Paz S., Rubio C., Gutiérrez A.J. (2021). Metals in commercial fish in the Galapagos marine reserve: Contribution to food security and toxic risk assessment. J. Environ. Manag..

[39] Jesus I.S., De Da Silva Medeiros R.L., Cestari M.M., De Almeida Bezerra M., De Mello Affonso P.R.A. (2014). Analysis of metal contamination and bioindicator potential of predatory fish species along Contas River basin in northeastern Brazil. Bull. Environ. Contam. Toxicol..

[40] Lima M.W., Pereira W.V.S., Souza E.S., Teixeira R.A., da Conceição Palheta D., Faial K.C.F., Costa H.F., Fernandes A.R. (2022). Bioaccumulation and human health risks of potentially toxic elements in fish species from the southeastern Carajás Mineral Province, Brazil. Environ. Res..

[41] Moiseenko T., Gashkina N. (2020). Distribution and bioaccumulation of heavy metals (Hg, Cd, and Pb) in fish: Influence of the aquatic environment and climate. Environ. Res. Lett..

[42] Majnoni F., Rezaei M., Mansouri B., Hamidian A.H. (2013). Metal concentrations in tissues of common carp, Cyprinus carpio, and silver carp, Hypophthalmichthys molitrix from the Zarivar Wetland in Western Iran. Arch. Pol. Fish..

[43] Peakall D., Burger J. (2003). Methodologies for assessing exposure to metals: Speciation, bioavailability of metals, and ecological host factors. Ecotoxicol. Environ. Saf..

[44] Nowosad J., Kucharczyk D., Szmyt M., Łuczynska J., Tamás M., Horváth L. (2021). Changes in cadmium concentration in muscles, ovaries, and eggs of Silver European Eel (Anguilla anguilla) during maturation under controlled conditions. Animals.

[45] Wirth M., Kirschbaum F., Gessner J., Krüger A., Patriche N., Billard R. (2000). Chemical and biochemical composition of caviar from different sturgeon species and origins. Nahrung.

[46] Has-Schön E., Bogut I., Strelec I. (2006). Heavy metal profile in five fish species included in human diet, domiciled in the end flow of River Neretva (Croatia). Arch. Environ. Contam. Toxicol..

[47] Anandkumar A., Nagarajan R., Prabakaran K., Bing C.H., Rajaram R. (2018). Human health risk assessment and bioaccumulation of trace metals in fish species collected from the Miri coast, Sarawak, Borneo. Mar. Pollut. Bull..

[48] Morcillo P., Esteban M.A., Cuesta A. (2017). Mercury and its toxic effects on fish. AIMS Environ. Sci..

[49] Bronzi P., Chebanov M., Michaels J.T., Wei Q., Rosenthal H., Gessner J. (2019). Sturgeon meat and caviar production: Global update 2017. J. Appl. Ichthyol..

[50] Sicuro B. (2019). The future of caviar production on the light of social changes: A new dawn for caviar?. Rev. Aquac..

[51] European Commission (EC) (2006). Commission Regulation No 1881/2006 of 19 December 2006 setting maximum levels for certain contaminants in foodstuffs (Text with EEA relevance). http://faolex.fao.org/docs/pdf/eur68134.pdf.

[52] Mansouri B., Maleki A., Davari B., Karimi J., Momeneh V. (2015). Estimation of target hazard quotients for heavy metals intake through the consumption of fish from Sirvan River in Kermanshah Province, Iran. J. Adv. Environ. Health Res..

[53] Esfahani N.B., Jafari M., Moravejolahkami A.R. (2020). Heavy metals concentration and target hazard quotients assessment through the consumption of fish muscle Ctenopharyngodon Idella (Cyprinidae) from markets in Ahvaz province, Iran. Nutr. Food Sci..

[54] Mukherjee J., Saha N.C., Karan S. (2021). Bioaccumulation pattern of heavy metals in fish tissues and associated health hazards in human population. Environ. Sci. Pollut. Res..

[55] Rezaei H., Zarei A., Kamarehie B., Jafari A., Fakhri Y., Bidarpoor F., Karami M.A., Farhang M., Ghaderpoori M., Sadeghi H. (2019). Levels, distributions and health risk assessment of lead, cadmium and arsenic found in drinking groundwater of Dehgolan’s Villages, Iran. Toxicol. Environ. Health Sci..

